# Towards the international interoperability of clinical research networks for rare diseases: recommendations from the IRDiRC Task Force

**DOI:** 10.1186/s13023-023-02650-4

**Published:** 2023-05-09

**Authors:** Rima Nabbout, Galliano Zanello, Dixie Baker, Lora Black, Isabella Brambilla, Orion J. Buske, Laurie S. Conklin, Elin Haf Davies, Daria Julkowska, Yeonju Kim, Thomas Klopstock, Harumasa Nakamura, Kim G. Nielsen, Anne R. Pariser, Jose Carlos Pastor, Maurizio Scarpa, Maureen Smith, Domenica Taruscio, Stephen Groft

**Affiliations:** 1 Department of Pediatric Neurology, Reference Center for Rare Epilepsies, Hôpital Necker-Enfants Malades, APHP, member of ERN EPICARE, Institut Imagine, INSERM U1163, Université Paris Cité, Paris, France; 2 Institut National de la Santé et de la Recherche Médicale, Paris, France; 3 Martin, Blanck, and Associates, Arlington, VA USA; 4 Sanford Research, Sioux Falls, SD USA; 5 Dravet Italia Onlus Italy – ePAG EpiCARE, Verone, Italy; 6 PhenoTips, Toronto, ON Canada; 7 ReveraGen BioPharma, Rockville, MD USA; 8 Aparito Limited, Metabolic Support UK, Wrexham, UK; 9 Korea Disease Control and Prevention Agency, Cheongju-si, Chungcheongbuj-do Korea; 10 Friedrich-Baur-Institute, Department of Neurology, LMU Klinikum, Ludwig-Maximilians-Universität München, Ziemssenstr. 1, 80336 Munich, Germany; 11 Department of Clinical Research Support, Clinical Research and Education Promotion Division, National Center of Neurology and Psychiatry, Tokyo, Japan; 12 Department of Paediatrics and Adolescent Medicine, Copenhagen University Hospital, Rigshospitalet, Copenhagen, Denmark; 13 Alltrna, Cambridge, MA USA; 14 IOBA (Eye Institute), University of Valladolid, Valladolid, Spain; 15 Regional Coordinating Center for Rare Diseases, Udine University Hospital, Udine, Italy; 16 European Reference Network. For Hereditary Metabolic Diseases (MetabERN), Dublin, Ireland; 17 Canadian Organization for Rare Disorders, Toronto, ON Canada; 18 National Centre for Rare Diseases, Istituto Superiore di Sanità, Rome, Italy; 19 Division of Rare Diseases Research Innovation, National Center for Advancing Translational Sciences, National Institutes of Health, Bethesda, MD USA

**Keywords:** Rare diseases, Clinical research networks, Interoperability, Patient unmet needs, Diagnosis, Therapies, IRDiRC

## Abstract

**Background:**

Many patients with rare diseases are still lacking a timely diagnosis and approved therapies for their condition despite the tremendous efforts of the research community, biopharmaceutical, medical device industries, and patient support groups. The development of clinical research networks for rare diseases offers a tremendous opportunity for patients and multi-disciplinary teams to collaborate, share expertise, gain better understanding on specific rare diseases, and accelerate clinical research and innovation. Clinical Research Networks have been developed at a national or continental level, but global collaborative efforts to connect them are still lacking. The International Rare Diseases Research Consortium set a *Task Force on Clinical Research Networks for Rare Diseases* with the objective to analyse the structure and attributes of these networks and to identify the barriers and needs preventing their international collaboration. The Task Force created a survey and sent it to pre-identified clinical research networks located worldwide.

**Results:**

A total of 34 responses were received. The survey analysis demonstrated that clinical research networks are diverse in their membership composition and emphasize community partnerships including patient groups, health care providers and researchers. The sustainability of the networks is mostly supported by public funding. Activities and research carried out at the networks span the research continuum from basic to clinical to translational research studies. Key elements and infrastructures conducive to collaboration are well adopted by the networks, but barriers to international interoperability are clearly identified. These hurdles can be grouped into five categories: funding limitation; lack of harmonization in regulatory and contracting process; need for common tools and data standards; need for a governance framework and coordination structures; and lack of awareness and robust interactions between networks.

**Conclusions:**

Through this analysis, the Task Force identified key elements that should support both developing and established clinical research networks for rare diseases in implementing the appropriate structures to achieve international interoperability worldwide. A global roadmap of actions and a specific research agenda, as suggested by this group, provides a platform to identify common goals between these networks.

**Supplementary Information:**

The online version contains supplementary material available at 10.1186/s13023-023-02650-4.

## Introduction

Rare diseases present unique challenges to the research community, as they are not limited to specific patient populations nor do they respect national, political, or geographical boundaries. Estimates of the number of rare diseases vary, but there is agreement that more than 7000 rare diseases affect approximately 350 million people in the world [1]. A molecular basis of disease is estimated in more than 10,000 genetic and acquired rare diseases [2, 3]. Many of the disorders are complex diseases affecting multiple systems and organs requiring multiple and interdisciplinary expert consultation, and collaboration in the diagnostic work-up and in the lifelong treatment. About a half of patients with a rare disease are children, with approximately 30% not living to the age of five [4]. Despite the tremendous efforts by research investigators, patients, patient groups, and the biopharmaceutical and medical devices industries, it can take 5 years or longer to receive an accurate diagnosis and the number of therapies remains very limited [5–7].

Numerous obstacles stand in the way of advances in the diagnosis and treatment of rare diseases. These include a lack of understanding of the heterogeneity and variability of many rare diseases, a lack of knowledge of the natural history of the diseases across the lifespan, and a variable progression having a large range (spectrum) of genotype–phenotype correlations or existing co-morbidities resulting in different responses to treatment. There are also a lack of appropriate biomarkers and accepted, objective clinical endpoints to measure safety and efficacy of new and repurposed compounds. In addition, the ability to make correct diagnosis is a major barrier to the identification of relatively larger patient populations required for clinical trial participation.

The development of Clinical Research Networks (CRNs) for rare diseases has created an essential reference point to compensate the problem of small and geographically dispersed patient populations. It also favoured the thematic grouping of diseases and the collaborative efforts of multiple stakeholders (patients, patient groups, researchers, health care providers, industry) and multi-disciplinary experienced teams to gain increased knowledge about specific rare diseases. However, global collaborative efforts to connect groups and networks often working on a national or regional (world regions) level are still missing. To stimulate a better understanding of the value and roles of CRNs to the rare diseases community, the International Rare Diseases Research Consortium (IRDiRC) established a Task Force with the objectives to analyze the existing ecosystem and structure of national and international CRNs, and to identify the barriers and needs preventing international collaboration of these networks. This manuscript exposes the work and results of the Task Force.

## Methods

The Task Force created a survey to characterize the CRNs attributes, identify the tools, resources, standards used to reach international interoperability and also understand the barriers preventing the networks from reaching this goal (Additional file 1). The survey was divided into several domains addressing: the demography of the CRNs, the sources and mechanisms of funding, the type of activities and research conducted at the CRNs, the barriers to research collaboration with other networks, the international interoperability structure of the CRNs, the infrastructures conducive to collaboration, and the evaluation of CRNs effectiveness (Additional file 1).

The survey was sent to 95 CRNs localized across the world in July/August 2020 and a reminder was sent in September 2020. These CRNs were pre-identified by the members of the Task force who established a list based on their expertise validated through the CRNs websites or direct interviews (Additional file 2). A total of 34 responses were received between August and September 2020. Although the response rate was not as high as we would expect it to be, we identified through a website search that the 34 networks represent over 1500 clinical research sites located worldwide (Additional file 2), thus offering an already significant representation for the current study. The Task Force analyzed the data provided by the respondents and identified key elements that should support both developing and established CRNs in creating the appropriate structure to advance towards international interoperability.

In this analysis, all the networks were assessed equally despite their different size including giving the same weight to the different networks in order to observe global trends regarding their attributes and needs.

## Main text

The objective of the Task Force is to provide the rare disease community with a better understanding of the existing CRNs structure and attributes; to identify the barriers and needs preventing their international interoperability; and to suggest recommendations on key elements, that if implemented, could support the development of global collaborative actions between networks. The findings and view of the Task Force are presented below.

### Demography of the CRNs

**Fig. 1:**
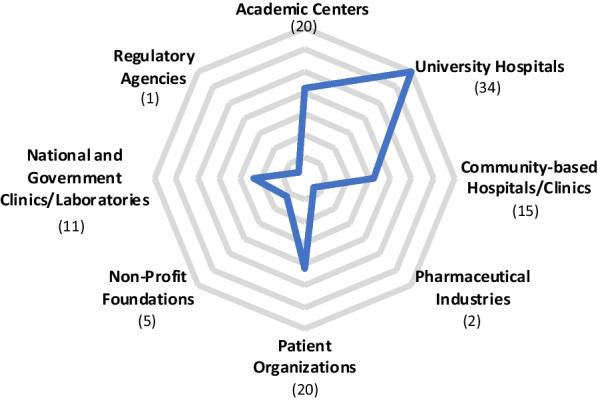
Member composition of the clinical research networks. The representation of each member within the 34 clinical research networks is indicated by the numbers in brackets

The list of the 34 CRNs including their year of creation and their geographical location is presented in Table 1. Sixteen of these are national and eighteen regional or international. Most of the CRNs have been created in countries or regions with a high Gross Domestic Product in the last two decades. The development of CRNs is linked with the recognition of rare diseases as a major public health problem by some national authorities and translate the importance of having central structures where patients and experienced multi-disciplinary teams can work together to better understand specific rare diseases and provide diagnostic and therapeutic options. Among the 34 listed CRNs, 30 are focusing on diseases that present in both paediatric and adult populations. As shown in Fig. 1, while it was expected to find that all of the 34 CRNs include hospitals in their members, academic centres and patient groups are also well recognized and integrated members, with both represented in 20 out of 34 CRNs. This finding highlights the role of patients as research partners and the ability for patients and their advocates to collaborate with multiple networks and capitalize on the synergy of their activities. On the other hand, pharmaceutical industries and regulatory agencies are poorly integrated into the composition of the 34 listed CRNs, even though clinical trials can be conducted at many of the responding research networks.

**Table 1:** List of clinical research networks included in the analysis

Name of the Network	Geography	Year of creation
Maternal Infant Child and Youth Research Network	Canada	2006
Care4Rare	Canada	2007
Canadian Inherited Metabolic Diseases Research Network	Canada	2012
Canadian Neuromuscular Disease network	Canada	2020
Children's Oncology Group	USA	2000
Rare Diseases Clinical Research Network	USA	2002
American Society of Clinical Oncology	USA	2010
NeuroNext	USA	2011
Undiagnosed Diseases Network	USA	2014
ESCAPE Clinical Research Network	Europe	1995
ERN CRANIO	Europe	2017
Endo-ERN	Europe	2017
EpiCARE	Europe	2017
ERKNet	Europe	2017
ERN-RND	Europe	2017
EURO-NMD	Europe	2017
ERN-EYE	Europe	2017
ERN GENTURIS	Europe	2017
EUROGEN	Europe	2017
MetabERN	Europe	2017
ERN RARE-LIVER	Europe	2017
ERN-RITA	Europe	2017
VASCERN	Europe	2017
NeuroSphinx-GBS	France	2014
AnDDI-Rares	France	2014
G2M	France	2015
NEOCYST	Germany	2016
Treat-ION	Germany	2019
Muscular Distrophy Clinical Trial Network	Japan	2012
Initiative on Rare and Undiagnosed Diseases	Japan	2015
Asia Pacific Society for Immunodeficiencies	Asia	2015
Asian Primary Immunodeficiency Network	Asia & Africa	2008
Collaborative International Neuromuscular Research Group	International	2000
Undiagnosed Diseases Network International	International	2014

The 34 clinical research networks included in the analysis are listed by geography and year of creation

### Activities, research and measures of effectiveness

**Fig. 2:**
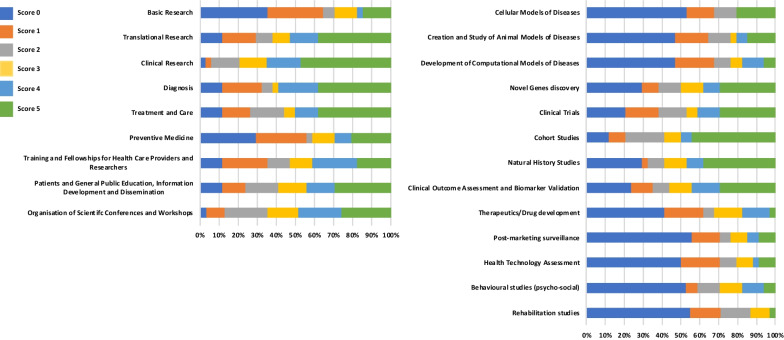
Activities and research conducted at the clinical research networks. The histograms represent the percentage of clinical research networks conducting various types of activities and research. The rating scale goes from score 0 (no activities) to score 5 (major activities)

As presented in Table 2 and Fig. 2, CRNs deliver multiple activities including research (from basic to clinical research), treatment and care, education and training, patient empowerment and organization of events, such as scientific conferences aimed to define a research agenda. This diversity of activities is representative of their role in gaining better understanding of rare diseases, translating these advances into more efficient clinical studies and better care for the patients, and disseminating this knowledge to the professional community, patients, caregivers and the public at large. However, we should also note that the type of activities conducted at the networks depends on their mandate, with some networks focusing mainly on clinical research and clinical trial readiness (e.g., US Rare Diseases Clinical Research Network). In contrast, other CRNs combine clinical research and care (e.g., European Reference Networks). A similar statement regarding the diversity of research conducted at the networks can be made (Table 2). CRNs present a strong research orientation towards the discovery of novel gene (scored 4–5 for 38% of the CRNs), clinical trials (41%), cohort studies (50%), natural history studies (47%) and validation of clinical outcome assessment and biomarkers (44%).

**Table 2:** Activities and research conducted at the clinical research networks

Activities conducted by the networks (% response)* Rating scale: 0* = *no activities, 5* = *major activities*	0	1	2	3	4	5
Basic Research	35	29	6	12	3	15
Translational Research	12	18	9	9	15	38
Clinical Research	3	3	15	15	18	47
Diagnosis	12	21	6	3	21	38
Treatment and Care	12	15	18	6	12	38
Preventive Medicine	29	27	3	12	9	21
Training and Fellowships for Health Care Providers and Researchers	12	24	12	12	24	18
Patients and General Public Education, Information Development and Dissemination	12	12	18	15	15	29
Organisation of Scientifc Conferences and Workshops	3	10	23	16	23	26

Research conducted by the networks (% response)* Rating scale: 0* = *no activities, 5* = *major activities*	0	1	2	3	4	5
Cellular Models of Diseases	53	15	12	0	0	21
Creation and Study of Animal Models of Diseases	47	18	12	3	6	15
Development of Computational Models of Diseases	47	21	9	6	12	6
Novel Genes discovery	29	9	12	12	9	29
Clinical Trials	21	18	15	6	12	29
Cohort Studies	12	9	21	9	6	44
Natural History Studies	29	3	9	12	9	38
Clinical Outcome Assessment and Biomarker Validation	24	12	9	12	15	29
Therapeutics/Drug development	41	21	6	15	15	3
Post-marketing surveillance	56	15	6	9	6	9
Health Technology Assessment	50	21	9	9	3	9
Behavioural studies (psycho-social)	53	6	12	12	12	6
Rehabilitation studies	55	16	16	10	0	3

*Percentage of clinical research networks conducting various types of activities and research. The rating scale goes from score 0 (no activities) to score 5 (major activities)*

Among the key performance indicators listed by the responders, the number of publications, number of patients recruited and participating in research, number of clinical research studies and clinical trials initiated, and the number of collaborations with other networks were highly valued and recognized as measures of effectiveness.

### International collaborative framework

**Fig. 3:**
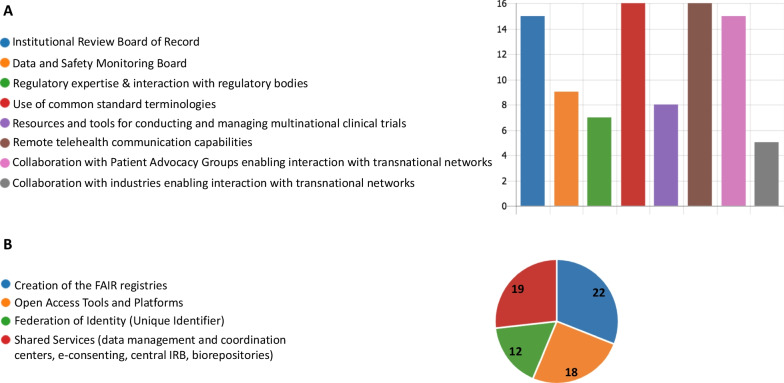
Key elements and infrastructures conducive to collaboration. The graphics represent the numbers of clinical research networks adopting various **A** key elements and **B** infrastructures to reach international interoperability

Establishing CRNs interoperability requires that the networks share common tools, ontologies, data standards, and procedures to facilitate their collaboration. Understanding the type of elements and infrastructures adopted by the CRNs to stimulate collaborative actions was an important objective of the Task Force. As shown in Fig. 3A, the elements established by the networks to reach international interoperability are diverse and address aspects linked to monitoring of clinical research and studies, management of multi-national clinical trials, collection and sharing of data, remote clinical assessment, collaboration with patient groups and industry, and interaction with regulatory bodies.

It is important to note that these elements are supported by the creation of specific infrastructures such as FAIR (**F**indability, **A**ccessibility, **I**nteroperability, and **R**euse of digital assets) data registries and open access platforms to find, share and re-use structured clinical data, central services to coordinate activities, and unique identifiers to pseudonymized personal patient data (Fig. 3B). Altogether, these findings confirm that CRNs have the potential to actively transform the rare disease research ecosystem not only by attracting multiple experienced stakeholders, but also by establishing the key elements and infrastructures supporting efficient management and coordination of activities, comprehensive data entry, collection, and sharing procedures in accordance with data standards and ontologies.

### Sustainability of the CRNs

**Fig. 4:**
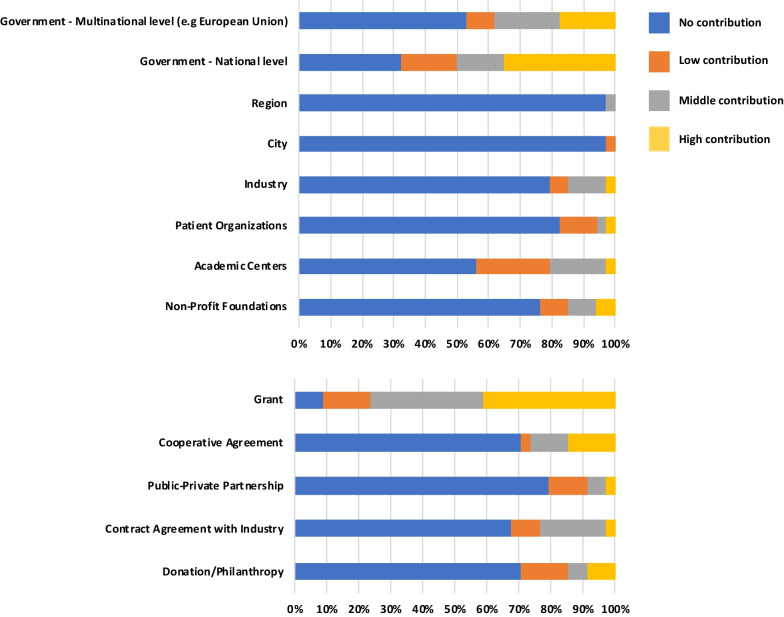
Sources and mechanisms of funding. The histograms represent the percentage of clinical research networks sustained through various sources and mechanisms of funding. The level of contribution for each funding source is presented on a scale going from no contribution to low, medium or high contribution. The same rating scale is used for the mechanisms of funding

Understanding how CRNs sustain their activities is critical, both in the need to clarify the funding contribution of the different stakeholders as well as to provide structural information for the developing networks. The sources and mechanisms of funding for the listed CRNs are presented in Table 3 and Fig. 4. Although diverse in their origins, the main funders appear to be national government (highest contribution for 35% of the CRNs, medium contribution 15%) or multi-national level (highest contribution 18%, medium contribution 21%). The mechanisms of funding demonstrated that public grants are the highest contribution by some margin (highest contribution 41%, medium contribution 35%) followed by cooperative agreements (highest contribution 15%, medium contribution 12%). In comparison, we found that contract agreement with industry (highest contribution 3%, medium contribution for 21%) and philanthropy (highest contribution 9%, medium contribution for 6%) contribute to a much lesser extent. It is interesting to note that differences regarding industry participation and contract agreements with industry are observable between different regions. For example, while some Asian and North American CRNs mentioned collaboration with and funding from industry, ERNs are not legal entities and cannot be funded by industry at the network level. Collaborations and clinical trials are therefore signed and managed at each clinical research site. Altogether, these results highlight the central role of public funders in sustaining CRNs activities either as a sole provider in Europe or as a main provider in other regions such as the United States.

**Table 3:** Sources and mechanisms of funding of CRNs

Sources of funding (% response)	No contribution	Low contribution	Medium contribution	High contribution
Government—Multinational level (e.g. European Union)	53	9	21	18
Government—National level	32	18	15	35
Region	97	0	3	0
City	97	3	0	0
Industry	79	6	12	3
Patient Organizations	82	12	3	3
Academic Centers	56	24	18	3
Non-Profit Foundations	77	9	9	6

Mechanisms of funding (% response)	No contribution	Low contribution	Medium contribution	High contribution
Grant	9	15	35	41
Cooperative Agreement	71	3	12	15
Public–Private Partnership	79	12	6	3
Contract Agreement with Industry	68	9	21	3
Donation/Philanthropy	71	15	6	9

Percentage of clinical research networks sustained through various sources and mechanisms of funding. The level of contribution for each funding source is presented on a scale going from no contribution to low, medium or high contribution. The same rating scale is used for the mechanisms of funding

### Barriers and needs preventing international collaboration

**Fig. 5:**
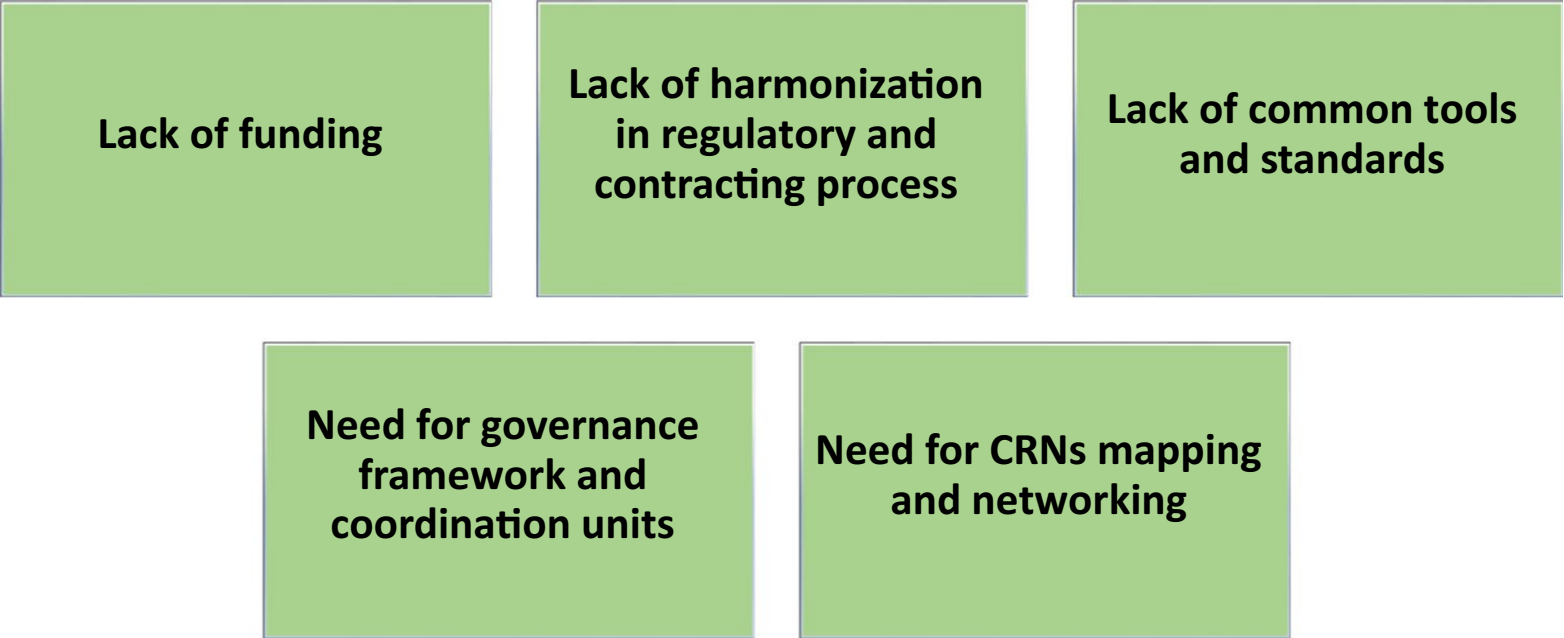
Hurdles preventing research collaboration between clinical research networks. The barriers and needs listed by the clinical research networks to reach international interoperability are grouped into five main categories

Despite the CRNs efforts to stimulate collaboration between networks, several barriers and needs preventing or slowing down collaborative actions at the international and supra-regional level have been highlighted. The barriers and needs listed by CRNs were grouped into five categories and are represented in Fig. 5.

The lack of funding has been repeatedly described to be a major obstacle and responders emphasized the importance to promote dedicated programmes involving both public and private funders to support international projects between networks. The lack of harmonization in regulatory and contracting process represents a second category. This includes restrictions associated with data sharing, concurrent IRB reviews, difference in national clinical trials guidance and procedure, different rules regarding collaboration with industry and intellectual property rights (i.e., some networks such as the ERNs not being authorized to collaborate with industry). The lack of common tools and data standards forms a third category in which the need for standardized common data elements, FAIR registries, and compatible data sharing platforms were mentioned multiple times. The fourth category highlights the needs for establishing a governance framework between the networks as well as the difficulties linked with the absence of central management and coordination units. Finally, the lack of CRNs mapping, which allows an easy identification of possible collaboration and networking, has been presented as another hurdle hampering global collaborative actions.

### Roadmap for the development of international CRNs for rare diseases

To stimulate and facilitate the development of interoperable networks, CRNs should engage further in networking and communication activities through the organization of conferences, workshops and webinars. Access to digital technologies including social media could provide great opportunities to support patient and researcher engagement in multiple networks. Continuous education, fellowship programmes and mentoring services across networks could also help health care providers, patient groups, and research investigators to expand knowledge and expertise during the lifespan of their career and build connections with other groups.

CRNs for rare diseases do not all have the same mandate, therefore, understanding the specificity of each network, defining common goals and the means to achieve them is essential. Shared tools and resources, harmonized standards (common data elements and ontologies) and guidelines (e.g., good data practice, FAIR data principles, clinical trial procedures), open access platforms, and data sharing agreements are central elements that can support the development of network international interoperability. Reaching an agreement on the implementation of these elements at the international level while preserving patient privacy and data will be critical to accelerate advances in rare disease research and address the unmet needs of patients across the world.

The public funds for national and regional plans for rare diseases, in addition to the positive experiences of private–public-partnership already established in some regions, could be a good example on how to expand these CRNs sharing a better know-how and increasing collaborative funding. In South Korea, one research program of supporting diagnosis for undiagnosed rare diseases patients [8], was expanded to a national initiative including government bodies, research institutes, and hospitals—aiming to collect genomic and medical information from million residents in South Korea including fifteen thousand of rare disease patients and their family members [9]. At the European level, the Innovative Medicines Initiative-funded connect4children project aims to create a sustainable pan-European collaborative paediatric network that will speed up and facilitate the development of high-quality clinical trials in children [10].

In this respect, development of CRNs for rare diseases in low- and middle-income countries should also be supported to ensure equity in access to affordable and effective treatment and care [11]. The RDI-WHO Collaborative Global Network for Rare Diseases aims to identify, assess, support and connect centres of expertise globally in order to leave no one behind [12].

### Future directions for CRNs

Existing and planned CRNs are well positioned to utilize the strengths of novel digital health technologies, data libraries with information gained from smart phones, mobile sensors, and remote monitoring devices. Information gained from artificial intelligence analyses and procedures to utilize Real World Data/Evidence from multiple sources and electronic health records could assist in the identification of possible new products for investigation or significant biomarkers to assess the safety and effectiveness of these interventions.

There are many novel approaches to improve patient access to medical care and clinical trials developed due to the spread of COVID-19 infections. Activities related to telemedicine appointments with increased reliance on Patient Reported Outcomes Measurements and Quality of Life assessments are all gaining increased traction and emphasis. Likewise, the introduction of decentralized clinical trials, e-Consent procedures, and growing acceptance of patient-entered data are all contributing to the patient-centricity and patient-focused drug development that is gaining rapid acceptance on a global basis. In many cases, this was already accepted and in place at sites of CRNs dedicated to rare diseases.

## Conclusions

Advancing towards the international interoperability of CRNs is crucial for gaining better understanding of rare diseases. It requires the establishment of key elements to support sharing of information and knowledge among multiple stakeholders and coordination of joint activities. Access to data from diverse patient populations and from multiple countries could profoundly expand interpretation capabilities of genomic sequencing results (pathogenic, benign, and variants of unknown significance) to assist in undiagnosed and all diseases. It would likely accelerate the identification of more accurate biomarkers, better define clusters of phenotypes, increase knowledge on the natural history of diseases, and facilitate patient engagement and collaboration in research. In doing so, it is essential to expand awareness, advocacy, and outreach to everyone—including those with lower socio-economic status, poor literacy, minority ethnic status, indigenous populations, and people living in underserved and marginalized urban and rural areas.

## Supplementary Information

**Additional file 1.** The survey developed to better understand the clinical research networks (CRN) activities and challenges included 37 questions in 9 parts addressing the demography of the CRN, the characterization of the CRN funds, the type of activities conducted, the types of research conducted, the barriers to research collaboration, the International interoperability structure of the CRN, the infrastructures conducive to collaboration and the methodology for the measurement of the key performance indicators.

**Additional file 2.** This table shows the number of clinical research sites and of health care providers institution for each CRN that answered the survey and that was included in this study. The search was performed on the websites of the CRNs in June-July 2022.

## Data Availability

The datasets analyzed as sources for the lists are available from the corresponding author upon request.

## Abbreviations

IRDiRC: International Rare Diseases Research Consortium
CRNs: Clinical Research Networks
ERNs: European Reference Networks
FAIR: Findability, accessibility, interoperability, and reuse of digital assets
RDI: Rare diseases international
WHO: World Health Organization
