# Supplementary material for: Towards the international interoperability of clinical research networks for rare diseases: recommendations from the IRDiRC Task Force
Source: Orphanet J Rare Dis. 2023 May 9;18:109. doi: 10.1186/s13023-023-02650-4 (PMC10169162; doi:10.1186/s13023-023-02650-4)
Supplement: Supplementary file 1 — Additional file 1. The survey developed to better understand the clinical research networks (CRN) activities and challenges included 37 questions in 9 parts addressing the demography of the CRN, the characterization of the CRN funds, the type of activities conducted, the types of research conducted, the barriers to research collaboration, the International interoperability structure of the CRN, the infrastructures conducive to collaboration and the methodology for the measurement of the key performance indicators. [file 13023_2023_2650_MOESM1_ESM.pdf]

IRDiRC Survey - Clinical Research Networks for Rare Diseases

This survey will be used to map and analyze the existing ecosystem of national and supranational clinical research networks. This survey will support IRDiRC in developing policy recommendations on guiding principles for an international framework of collaboration of these networks in respect to best practices, interoperability, tools and common goals.
The survey includes 37 questions and the average time for completion is 25 minutes.
Your personal information and data are collected by Inserm which is responsible for the Scientific Secretariat of IRDiRC.
Your personal information and data are collected according to the European General Data Protection Regulation rules.
If you require further details, please contact scisec-irdirc@ejprarediseases.org

* Required

I. Identification of the person completing the survey

1. First name *

2. Last name *

3. Email address *

4. Affiliation (e.g. Institution name) *

II. Demography of the Clinical Research Network

5. Name of the network *

6. What is the geography/location of the network? *

Please indicate the country of the network or its region (e.g. Europe) if the network is multi-national.

7. In which country is the network coordinating center located? *

8. In which year was the network created? *

9. Describe the primary medical domain of the network. Check all that apply. *

- [ ] Cardiology
- [ ] Dermatology
- [ ] Ear, Nose, Throat
- [ ] Endocrinology
- [ ] Gastroenterology
- [ ] Haematology
- [ ] Hepathology
- [ ] Immunology
- [ ] Inherited genetic disorders
- [ ] Metabolism
- [ ] Nephrology
- [ ] Neurology
- [ ] Oncology
- [ ] Ophtalmology
- [ ] Osteology
- [ ] Pulmonology
- [ ] Psychiatry
- [ ] Rhumatology
- [ ] Transplantation
- [ ] Urology
- [ ] Vascular disorders

10. If you wish, please specify the sub-domain of the network (e.g. rare epilepsy).

11. Is the network focus on one disease or a group/cluster of diseases? *

- ◯ One disease
- ◯ Group/Cluster of diseases

12. Is the network focus on pediatric or adult disease(s)? *

- ◯ Pediatric
- ◯ Adult
- ◯ Pediatric and Adult

13. What are the type of network members? Check all that apply. *

- ☐ Academic Centers (other than hospitals)
- ☐ University Hospitals
- ☐ Community-based Hospitals/Clinics
- ☐ Pharmaceutical Industries
- ☐ Patient Organizations
- ☐ Non-Profit Foundations
- ☐ National and Government Clinics/Laboratories
- ☐ Regulatory Agencies

14. If other types of network members are involved, please identify them below.

III. Characterization of the Clinical Research Network fund-

15. Identify the sources of funding based on their level of contribution. *

	No Contribution	Low Contribution	Medium Contribution	High Contribution
Government - Multinational level (e.g European Union)	○	○	○	○
Government - National level	○	○	○	○
Region	○	○	○	○
City	○	○	○	○
Industry	○	○	○	○
Patient Organizations	○	○	○	○
Academic Centers	○	○	○	○
Non-Profit Foundations	○	○	○	○

16. If other sources of funding are provided, please identify them below (describe in brackets their level of contibution).

17. Identify the mechanisms under which the network seeks for complementary funding based on their level of contribution. *

A public-private partnership is defined as a collaboration between a public institution and a private-sector company that can be used to finance, build, and operate projects.

	No Contribution	Low Contribution	Medium Contribution	High Contribution
Grant	○	○	○	○
Cooperative Agreement	○	○	○	○
Public-Private Partnership	○	○	○	○
Contract Agreement with Industry	○	○	○	○
Donation/Philanthropy	○	○	○	○

18. If other funding mechanisms are used, please identify them below (describe in brackets their level of contibution).

IV. Type of activities conducted at the Clinical Research

19. Describe the activities developed by your network and use the score scale to rate these activities. *

This rating scale goes from 0 to 5 with 0 representing no activity performed by the network and 5 representing major activities. . Basic Research aims at understanding the fundamental biological mechanims in health or disease. . Translational Research focuses on bench to bedside activities (development of diagnosis and/or therapies). . Clinical Research involves Human subjects or uses Human tissue or data to understand health and disease.

	0	1	2	3	4	5
Basic Research	○	○	○	○	○	○
Translational Research	○	○	○	○	○	○
Clinical Research	○	○	○	○	○	○
Diagnosis	○	○	○	○	○	○
Treatment and Care	○	○	○	○	○	○
Preventive Medicine	○	○	○	○	○	○
Training and Fellowships for Health Care Providers and Researchers	○	○	○	○	○	○
Patients and General Public Education, Information Development and Dissemination	○	○	○	○	○	○
	0	1	2	3	4	5
Organisation						

of Scientifc Conferences and Workshops ◯ ◯ ◯ ◯ ◯ ◯

20. If other activities are conducted within the network, please identify them below and assign them a score (in brackets next to the described activity).

V. Type of research conducted at the Clinical Research

21. Describe the research conducted by your network and use the score scale to rate these research activities. *

This rating scale goes from 0 to 5 with 0 representing no activity performed by the network and 5 representing major activities.

	0	1	2	3	4	5
Cellular Models of Diseases	○	○	○	○	○	○
Creation and Study of Animal Models of Diseases	○	○	○	○	○	○
Development of Computational Models of Diseases	○	○	○	○	○	○
Novel Genes discovery	○	○	○	○	○	○
Clinical Trials	○	○	○	○	○	○
Cohort Studies	○	○	○	○	○	○
Natural History Studies	○	○	○	○	○	○
Clinical Outcome Assessment and Biomarker Validation	○	○	○	○	○	○
Therapeutics/ Drug development	0	1	2	3	4	5

development

Post-marketing surveillance	○	○	○	○	○	○
Health Technology Assessment	○	○	○	○	○	○
Behavioural studies (psycho-social)	○	○	○	○	○	○
Rehabilitation studies	○	○	○	○	○	○

22. If other research activities are conducted within the network, please identify them below and assign them a score (in brackets next to the described activity).

VI. Barriers to research collaboration

23. What are the major obstacles encountered by your network for international collaboration with other clinical research networks? *

24. Which measures could be taken to facilitate international collaboration between clinical research networks? *

25. Select the barriers identified by your network that prevent research collaboration with academic institutions. Check all that apply. *

The Institutional Review Board (IRB) is an administrative body established to protect the rights and welfare of human research subjects participating in research activities conducted under the auspices of the institution with which the IRB is affiliated. The IRB has the authority to approve, disapprove, monitor, and require modifications in all research activities that fall within its jurisdiction as specified by both the federal regulations and institutional policy.

- [ ] Institutional Review Board/Ethical Review Board (e.g. concurrent IRB reviews required from multiple institutions for multi-site studies)
- [ ] Lack of funding and/or competition for limited research funding
- [ ] Lack of or limited collaborative models (including data sharing models)
- [ ] Lack of suitable connection with other research teams
- [ ] Lack of access to patients
- [ ] Intellectual property rights
- [ ] Publication rights
- [ ] Product manufacturing

26. If other barriers preventing collaboration with academic institutions are identified, please specify them below.

27. Select the barriers identified by your network that prevent research collaboration with industry. Check all that apply. *

The Institutional Review Board (IRB) is an administrative body established to protect the rights and welfare of human research subjects participating in research activities conducted under the auspices of the institution with which the IRB is affiliated. The IRB has the authority to approve, disapprove, monitor, and require modifications in all research activities that fall within its jurisdiction as specified by both the federal regulations and institutional policy.

- [ ] Institutional Review Board/Ethical Review Board (e.g. concurrent IRB reviews required from multiple institutions for multi-site studies)
- [ ] Lack of funding and/or competition for limited research funding
- [ ] Lack of or limited collaborative models (including data sharing models)
- [ ] Lack of suitable connection with other research teams
- [ ] Lack of access to patients
- [ ] Intellectual property rights
- [ ] Publication rights
- [ ] Product manufacturing
- [ ] Not applicable (if the network is not seeking to collaborate with industry)

28. If other barriers preventing collaboration with industry are identified, please specify them below.

VII. International interoperability structure of the Clinical Research Network

International interoperability is defined as the identification and implementation of common goals and use of compatible resources, tools, standards and/or policy elements to enable data sharing

29. Does your network plan to reach international interoperability? *

- ○ Yes
- ○ No

30. If affirmative, select the needs identified by your network to reach international interoperability. Check all that apply.

- ☐ Identification of and agreement on common goals with other networks
- ☐ Identification of common tools and resources that can be shared (e.g. accessible registries, secured data sharing platforms, common consent forms)
- ☐ Use of common standard terminologies (e.g. CDISC, HL7 FHIR, SNOMED, ICD, OrphaCodes)
- ☐ Common governance framework
- ☐ Sharing best practices and mentoring
- ☐ Institutional Review Board (IRB) of Record (special agreement between two or more institutions who are engaged in multi-site human subjects research)
- ☐ Data and Safety Monitoring Board (special agreement between two or more institutions who are engaged in multi-site human subjects research)
- ☐ Regulatory expertise
- ☐ Remote telehealth communication capabilities
- ☐ Collaboration with Patient Advocacy Groups enabling interaction with transnational networks
- ☐ Collaboration with industries enabling interaction with transnational networks

31. If other needs have been identified by your network to reach international interoperability, please describe them below.

32. Select the key elements already established by your network to reach international interoperability. Check all that apply.

- ☐ Institutional Review Board (IRB) of Record (Special agreement between two or more institutions who are engaged in multi-site human subjects research)
- ☐ Data and Safety Monitoring Board (Special agreement between two or more institutions who are engaged in multi-site human subjects research)
- ☐ Regulatory expertise and interaction with regulatory bodies
- ☐ Use of common standard terminologies (e.g. CDISC, HL7 FHIR, SNOMED, ICD, HPO, OrphaCodes, etc)
- ☐ Resources and tools for conducting and managing multi-national clinical trials
- ☐ Remote telehealth communication capabilities
- ☐ Collaboration with Patient Advocacy Groups enabling interaction with transnational networks
- ☐ Collaboration with Bio-Pharmaceutical, Medical Devices and/or Sequencing industries enabling interaction with transnational networks
- ☐ None of them

33. If other key elements have been established by your network to reach international interoperability, please identify them below.

VIII. Infrastructures conducive to collaboration

34. What types of infrastructure conducive to collaboration have been adopted by your network? Check all that apply. *

- [ ] Creation of the FAIR registries (data stored in databases should be Findable, Accessible, Interoperable and Reusable to allow data exchange)
- [ ] Open Access Tools and Platforms
- [ ] Federation of Identity (Unique Identifier)
- [ ] Shared Services (data management and coordination centers, e-consenting, central IRB, biorepositories)

35. If other types of infrastructure conducive to collaboration were developed by your network, please identify them below.

IX. Measurement of the Clinical Research Network

36. What are the key performance/research indicators that are used by your network to measure its effectiveness? Check all that apply. *

- [ ] Number of publications
- [ ] Publication impact factor
- [ ] Number of patents
- [ ] Number of collaborations with other networks
- [ ] Number of webinars, educational activities, training courses, conference and meetings organized
- [ ] Number of patients recruited and participating in research studies
- [ ] Number of clinical research studies initiated (involving Human subjects)
- [ ] Number of novel genes discovered
- [ ] Number of clinical trials initiated
- [ ] Number of clinical research studies completed (involving Human subjects)
- [ ] Number of clinical trials completed
- [ ] Number of clinical trials leading to an approved product by a regulatory agency

37. If other key performance indicators were developed by your network, please identify them below.

This content is neither created nor endorsed by Microsoft. The data you submit will be sent to the form owner.

Microsoft Forms
